# Brown Dog Tick (*Rhipicephalus sanguineus* Sensu Lato) Infection with Endosymbiont and Human Pathogenic *Rickettsia* spp., in Northeastern México

**DOI:** 10.3390/ijerph19106249

**Published:** 2022-05-20

**Authors:** Jordan Salomon, Nadia Angelica Fernandez Santos, Italo B. Zecca, Jose G. Estrada-Franco, Edward Davila, Gabriel L. Hamer, Mario Alberto Rodriguez Perez, Sarah A. Hamer

**Affiliations:** 1 Ecology and Evolutionary Biology Program, Texas A&M University, College Station, TX 77843-2475, USA; jordansalomon@tamu.edu; 2 Instituto Politécnico Nacional, Centro de Biotecnología Genómica, Reynosa 88710, Mexico; nfernandezs@ipn.mx (N.A.F.S.); jestradaf@ipn.mx (J.G.E.-F.); 3 Department of Entomology, Texas A&M University, College Station, TX 77843-2475, USA; ghamer@tamu.edu; 4 Department of Veterinary Integrative Biosciences, Texas A&M University, College Station, TX 77843-2475, USA; ibzecca@cvm.tamu.edu (I.B.Z.); edavila@cvm.tamu.edu (E.D.)

**Keywords:** *Rickettsia parkeri*, *Rhipicephalus sanguineus*, dogs, tick-borne disease, rickettsiosis

## Abstract

Of the documented tick-borne diseases infecting humans in México, Rocky Mountain spotted fever (RMSF), caused by the Gram-negative bacterium *Rickettsia rickettsii*, is responsible for most fatalities. Given recent evidence of brown dog tick, *Rhipicephalus sanguineus* s.l., as an emerging vector of human RMSF, we aimed to evaluate dogs and their ticks for rickettsiae infections as an initial step in assessing the establishment of this pathosystem in a poorly studied region of northeastern México while evaluating the use of dogs as sentinels for transmission/human disease risk. We sampled owned dogs living in six disadvantaged neighborhoods of Reynosa, northeastern México to collect whole blood and ticks. Of 168 dogs assessed, tick infestation prevalence was 53%, composed of exclusively *Rh. sanguineus* s. l. (*n* = 2170 ticks). Using PCR and sequencing, we identified an overall rickettsiae infection prevalence of 4.1% (*n* = 12/292) in ticks, in which eight dogs harbored at least one infected tick. Rickettsiae infections included *Rickettsia amblyommatis* and *Rickettsia parkeri*, both of which are emerging human pathogens, as well as *Candidatus* Rickettsia andeanae. This is the first documentation of pathogenic *Rickettsia* species in *Rh. sanguineus* s.l. collected from dogs from northeastern México. Domestic dog infestation with *Rickettsia*-infected ticks indicates ongoing transmission; thus, humans are at risk for exposure, and this underscores the importance of public and veterinary health surveillance for these pathogens.

## 1. Introduction

Among the bacterial zoonoses, the Gram-negative rickettsiae are the most common vector-borne pathogens [1] and are the cause of the majority of human deaths in North America [2]. The most common tick-borne pathogenic *Rickettsia* species in the Americas include *R. rickettsii*, *R. parkeri*, and *R. africae* [3]. Tick vectors of these pathogens are distributed globally, and vector species in the Americas include *Dermacentor variabilis*, *Amblyomma maculatum*, and *Rh. sanguineus* s. l. [4]. Whereas *D. variabilis* and *A. maculatum* are generalist blood-feeders, *Rh. sanguineus* s. l. primarily feed on dogs during all life stages, while occasionally feeding on humans or other animals [5,6].

Lyme disease, ehrlichiosis, and spotted fever group rickettsiosis (SFGR), including Rocky Mountain spotted fever (RMSF), have been reported in México [7,8,9,10,11], with RMSF being the most prevalent and fatal tick-borne disease in the country [12,13]. Rocky Mountain spotted fever is especially devastating in México as the majority of mortalities are children [14,15]. The tick-borne rickettsial zoonoses manifest similarly in human clinical diagnoses and symptoms; however, they are caused by genetically distinct bacteria species with differing ecologies [2,16,17]. The distribution and prevalence of tick-borne pathogenic *Rickettsia* spp. in animals and ticks throughout México is not well defined [18]. Furthermore, laboratory diagnostics of human rickettsiosis is challenging, which complicates the treatment of patients and the implementation of public health policy.

Across México, rickettsiae pathogens have been described in vectors and hosts in northern Baja California, Sonora, Chihuahua [19], Campeche [20,21], Yucatán [22], Tabasco [23], Veracruz [21,24], Tamaulipas [24,25,26], and Coahuila [4,12,27]. Ongoing outbreaks of RMSF since 2008, with high fatality rates, in northwestern regions of México have been much of the focused surveillance, with human disease also reported from adjacent indigenous communities of Arizona [28,29,30,31,32]. Studies have identified *R. rickettsii*, *R. amblyommatis* (formerly known as *R. amblyommii* or *Candidatus R. amblyommii* [33]), and *Rh. rhipicephalus* s. l. from ticks removed from humans, dogs, deer, bobcats, and cattle within the state of Tamaulipas [24,25,26], and the region is predicted highly suitable for *R. parkeri* to exist [34], yet there have been no reports of human tick-borne rickettsial diseases in this region nor highly urbanized neighborhoods.

Dogs are commonly involved in human *Rickettsia* outbreaks, as they are integrated into human communities and support tick populations [28,29,30,32,35,36]. The widespread nature of *Rh. sanguineus* s. l. on dogs and the ubiquity of dogs within human domiciles suggest that the routine surveillance of these ticks on dogs can provide useful information for both veterinary and human health risk assessments [15,30,35,37]. Here, we sampled *Rh. sanguineus* s. l. from dogs in predominantly low-income neighborhoods of northeastern México. Our objectives were to (i) describe the infestation prevalence of ticks on privately owned dogs across six neighborhoods in relation to dog demographic data; and (ii) characterize tick infection prevalence with *Rickettsia* species.

## 2. Methods

### 2.1. Sample Collection

**Figure 1:**
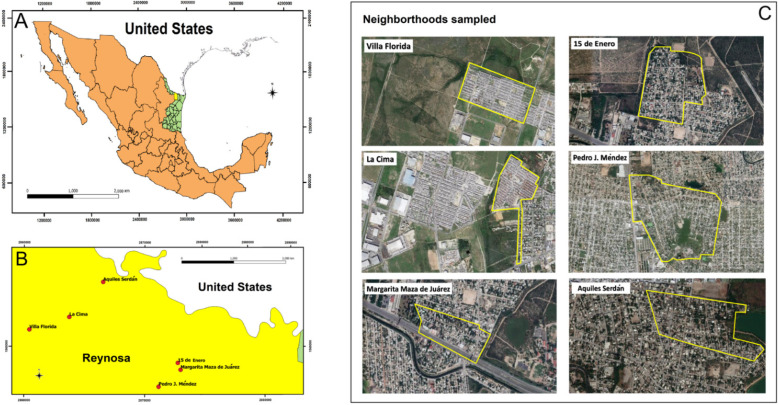
(**A**) Green area shows the State of Tamaulipas in northeastern México. (**B**) Red dots show the locations of the neighborhoods that were sampled within the city of Reynosa, in the State of Tamaulipas. (**C**) Satellite images show the land use composition of the six sampled neighborhoods in Reynosa. Figures are original maps, created by the authors using QGIS 3.18.2 with public domain map data from INEGI, and satellite images from Google maps. (https://qgis.org/en/site/) (accessed on 15 September 2021) with public domain map data from INEGI (https://www.inegi.org.mx/app/mapas/) (accessed on 15 September 2021), and satellite images from Google maps (https://www.google.com.mx/maps) (accessed on 15 September 2021).

We sampled dogs in six different neighborhoods within Reynosa, Tamaulipas of northeastern México (Figure 1) between 4 April through to 31 August 2019, as the summer season is representative of historical RMSF case reports [12]. These neighborhoods included: Aquiles Serdán (26.09, −98.31), Pedro J. Méndez (26.01, −98.27), Margarita Maza de Juárez (26.03, −98.25), 15 de Enero (26.03, −98.25), Villa Florida (26.06, −98.38), and La Cima (26.07, −98.34), as previously described [38]. Each neighborhood was selected based on being considered either of low or low–medium socioeconomic status (Table S1) and the available support of the local health neighborhood committees as the homes within these neighborhoods are built with weak infrastructures to minimize costs and development times. These neighborhoods typically have little veterinary care for their owned dogs, as well as large populations of stray dogs. All neighborhoods were sampled once, except for the neighborhood 15 de Enero, which was sampled once in May and once in June. Dogs were enrolled during neighborhood visits usually in a centralized home provided by a health neighborhood member, in empty nearby lots, and in door-to-door visits. As an incentive for participation, free rabies vaccinations were provided as a public health protective measure. Each dog was inspected for ticks, which were removed with forceps and placed into 70% ethanol. Blood was collected into EDTA tubes. Ticks and blood were exported to Texas A&M University for processing. From each dog, basic demographics were obtained including sex and age, and an estimation of breed and age was provided by the owner.

### 2.2. Tick Identification

**Figure 2:**
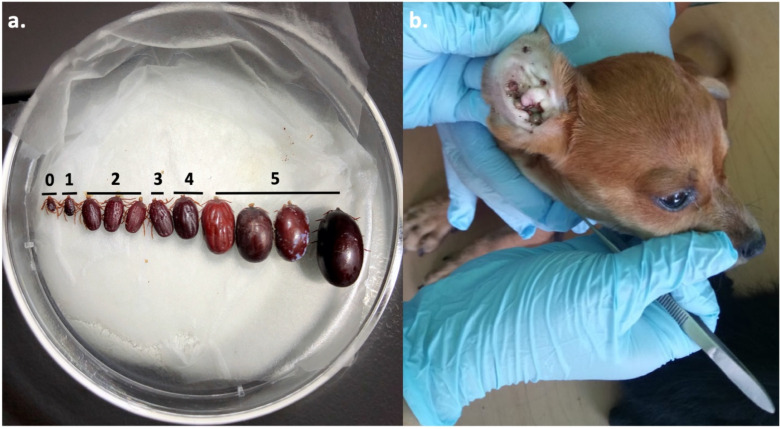
(**a**) Ticks were scored for engorgement on a scale from 0 to 5. This image is an example of the scoring scale for *Rh. sanguineus* s. l. adult females. Each life stage was scaled appropriately, except males were grossly indistinguishable and were therefore not scored for engorgement. (**b**) Removing ticks from participating dog ears upon inspection. This individual Chihuahua had 526 *Rh. sanguineus* s. l. attached.

All ticks were identified in terms of species, life stage, and sex, under a dissecting microscope (Furman and Loomis 1998). Representative ticks of each life stage and sex were submitted as voucher specimens to the Texas A&M Insect Collection of the Department of Entomology (Accession No. X1689674), with the collection information of these voucher specimens also being submitted to the open-access Global Biodiversity Information Facility data source. We scored the engorgement status of ticks on a scale of 0–5, in which a 0 was used for flat ticks with no appreciable bloodmeal, whereas a 5 was extremely engorged and presumed to be near repletion (Figure 2a). All adult male ticks appeared flat and were not given an engorgement status.

### 2.3. DNA Extractions

A stratified random subset (292 of 2170) of ticks was selected for molecular analysis. After tallying the number of ticks collected per dog (burden), a minimum of 20% of each dog’s tick burden was selected for DNA extraction, up to a maximum of 10 ticks on a dog with 200 or more ticks present. The first selection stratum was tick life stage; due to the rarity of immature ticks in the sample set, larvae and nymphs were always selected for processing when present. The second selection stratum was engorgement score, and those with higher engorgement scores were selected for processing over flat ticks to better represent any pathogens circulating in the dog’s blood. Each individual tick was sliced repeatedly using a sterile number 11 scalpel blade and then subjected to DNA extraction using a commercially available kit (E.Z.N.A Tissue DNA Kit; Omega Bio-Tek, Norcross, GA, USA) and overnight incubation for lysis, with a two-step final elution bringing the final volume to 50 μL. For any dog that had one or more *Rickettsia*-positive tick, we subsequently extracted DNA from 50 μL of whole dog blood using this same extraction kit, in which the incubation time for lysis was 10 min. In the case of a tick found positive for a pathogenic SFGR, all remaining ticks from that dog that did not meet the initial selection criteria were then processed in full.

### 2.4. PCR for the Genus Rickettsia and DNA Sequencing

To test for the presence of *Rickettsia* species in hard ticks [16,17,39,40] within each extracted tick, we adapted the semi-nested protocol from Wikswo et al. 2008 [41] to amplify the ompA gene of *Rickettsia,* a protein important in pathogenesis and a common target for detecting several species of SFG *Rickettsia* [42]. To reduce the potential for PCR inhibitory effects of hemoglobin [43], we added 1µL of 1 mg/mL bovine serum albumin (final PCR concentration of 0.04 µM) for every reaction [41]. Further alterations of the established PCR protocol included using touchdown thermocycling to minimize non-target amplification [44] with FailSafe™ 2X PCR Premix E and PCR enzyme (Lucigen, Middleton, WI, USA). PCR conditions were an initial pre-denature step for 1 min at 95 °C. Then, 10 cycles of amplification occurred, with a denature cycle running for 30 s at 95 °C followed by the touchdown annealing step starting at 56.5 °C and increasing by 0.1 °C until 57.5 °C for 30 s each followed by 20 cycles of annealing at 56.9 °C. An extension cycle was run at 72 °C for 1 min. Finally, an elongation step was run at 72 °C for 5 min. This was immediately followed with a semi-nested assay. The only changes in the semi-nested assay were during the touchdown annealing steps, for which 10 cycles were run, each starting at 59 °C and increasing by 0.1 °C until 60 °C for 30 s followed by 30 cycles of annealing at 60 °C for 30 s each cycle. Every PCR reaction used an SFGR positive control [45,46] and a negative control of PCR water. Prior to establishing this PCR protocol, we used up to five published PCR protocols targeting different genes (Table 1). However, these protocols produced multiple bands of variable fragment sizes per reaction, which sequenced to dog DNA or tick DNA and therefore were not used to generate data in the current study.

All PCR products were visualized via gel electrophoresis, and resulting amplicons were purified with ExoSAP-IT (Affymetrix, Santa Clara, CA, USA). Bidirectional Sanger sequencing was performed (Eton Bioscience Inc., San Diego, CA, USA). In Geneious (v 9.1.8), the forward and reverse sequences were trimmed, edited, and aligned to determine a consensus sequence which was compared to published sequences in NCBI GenBank [52]. Our criteria for concluding a sample as positive and identifying the *Rickettsial* spp. included a distinct band of approximately 550 bp with a sequence at least 97% identical to a published sequence (Table 2). Sequences were submitted to NCBI GenBank (accession numbers of OM743005-OM743016).

### 2.5. Statistical Analysis

We tested for differences in the mean tick burdens (mean number of ticks attached per dog) among dogs from the different neighborhoods and between dog sexes using the Kruskal–Wallis rank sum test, followed by a Dunn’s post hoc test. These calculations were run with R Version 1.2.5042 using the ‘*dunn.test*’ and ‘*stats*’ packages [53,54]. Generalized linear mixed models (GLMMs) with negative binomial error distribution and neighborhood as a random variable were used to determine the effect of dog sex and estimated age (continuous data ranging from 1 month to 10 years) on the outcome of tick burden. Similarly, GLMMs with a binomial error distribution and neighborhood as a random effect were used to determine whether the effects of the ticks’ life stage, ticks’ level of engorgement, tick burden of a host, host age, and host sex had any interaction on the outcome of a tick harboring rickettsiae. Lastly, a GLM with binomial error distribution was used to test the effect neighborhood had on the probability of identifying a tick positive for Rickettsia. These models were calculated with the ‘*lme4*’ and ‘*MASS*’ packages [55,56]. Models with multiple predictor variables were checked for multicollinearity using the ‘*vif*’ function within the ‘*car*’ package [57], and predictor variables with variance of inflation factors 5 or greater were either excluded from the models or set as a random variable.

## 3. Results

### 3.1. Sample Collection

Overall, 168 dogs were enrolled in this study across six neighborhoods (collection sites) in Reynosa, northeastern México. Dog enrollment varied by neighborhood, where the most enrolled was 45 dogs from La Cima, and the least enrolled was 9 dogs from Margarita Maza de Juárez (Table 3). The sex ratio was nearly equal (females, *n* = 83; males, *n* = 81; unknown, *n* = 4). Throughout the six neighborhoods, the average age of the dogs sampled was three years, with an age range of one month to fourteen years. Eighteen different dog breeds or mixes were recorded, with 50% (*n* = 84) of them described as mixed, and 24% (*n* = 40) were Chihuahuas.

**Figure 3:**
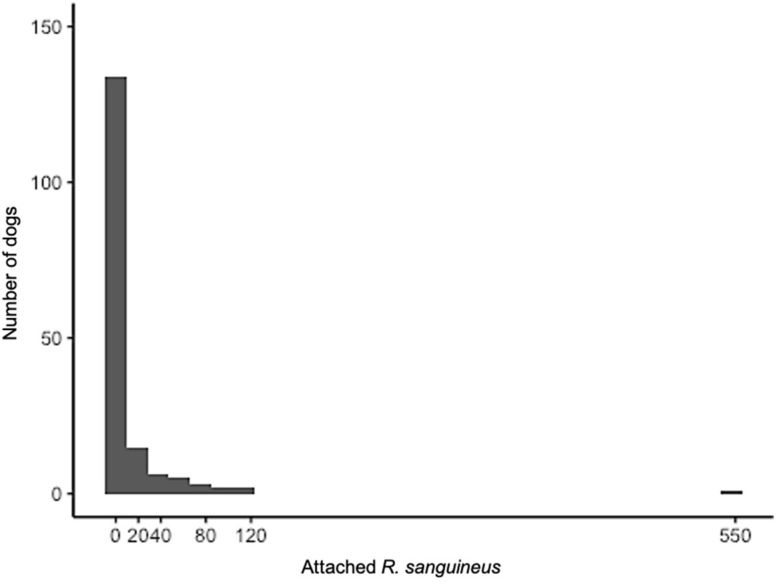
Tick burdens of those dogs from Reynosa, northeastern México were highly skewed, where most dogs had no ticks, but one had over 500 ticks.

**Figure 4:**
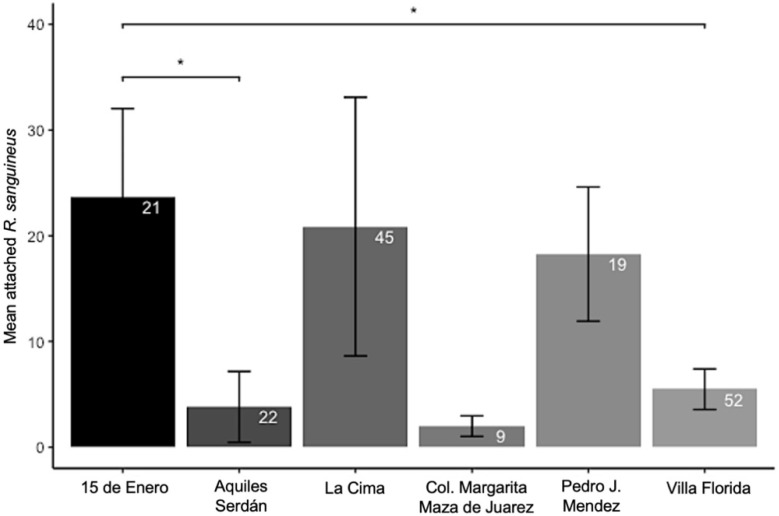
Mean attached *Rh. sanguineus* on dogs at each collection site. Error bars represent the standard error of the mean. The white number inside the bars represent how many dogs were enrolled in the study at each neighborhood. Significant differences are indicated as “*”, where *p* < 0.05.

A total of 89 of 168 dogs harbored at least one tick for an overall infestation prevalence of 53% (Table 3). A total of 2170 ticks were collected from the 89 infested dogs (Figure 3). Across all neighborhoods, the average tick burden was 13 ticks per dog (*n* = 168; ±47 SD), with the largest tick burden of 546 ticks attached to a Chihuahua dog (Figure 2b). Mean tick burdens were significantly different across neighborhoods (Kruskal–Wallis chi-square test = 17.02, df = 5, *p*-value = 0.005). Dogs living in 15 de Enero (23.7 ± 8.4 SE) had significantly greater mean tick burdens than those of Aquiles Serdán (3.8 ± 0.97 SE, *p*-value = 0.02) and Villa Florida (Figure 4; 5.5 ± 1.9 SE, *p*-value = 0.02). Dog age (*p*-value = 0.64) and sex (*p*-value = 0.16) were not predictive of tick burden (Table S2).

All ticks identified from dogs were *Rh. sanguineus* (*n* = 2149, Table 4). There was a total of 21 ticks (<1%) that were unidentifiable due to poor condition or missing anatomic parts. Of those for which life stage was assigned, 50% were adults (*n* = 1074), 40% were nymphs (*n* = 866), and 10% were larvae (*n* = 217). Adults were 58% male and 42.0% female. The average engorgement score of adult females was 2.1, for nymphs it was 3.0, and for larvae it was 3.0 (Table 4).

### 3.2. Molecular Testing for Rickettsiae

Two-hundred ninety-two individual ticks met the selection criteria and were tested for rickettsiae. Overall, there was a 4.1% infection prevalence for rickettsiae (*n* = 12/292) through the amplification of the ompA gene via the touchdown PCR protocol followed by sequencing to identify the genospecies. *Candidatus* R. andeanae was the most common (*n* = 8/12), followed by *R. amblyommii* (*n* = 3/12) and a single tick with *R. parkeri* (Table 3). Of the nine dogs which had *Rickettsia*-positive ticks, all dog blood samples tested negative for rickettsiae. GLMM analyses of the individual ticks’ life stage (*p*-values = 0.32 and 0.78), level of engorgement (*p*-value = 0.39), and the dog tick burden (*p*-value = 0.47), had no significant effects on the outcome of tick infection (Table S3). Younger dogs (*p*-value = 0.03) and male dogs (*p*-value = 0.02) were found to be less likely to be associated with harboring a *Rickettsia*-positive tick. There was no significant association between the neighborhood of collection and the outcome of tick infection (Table S4).

The *R. parkeri*-positive tick was attached to a two-year old female, mixed-breed dog from the neighborhood Pedro J. Méndez. This was the only infected tick among the total of 22 ticks on the dog (4 ticks were processed in the initial stratified random screening; the remaining 18 were processed following the finding of a pathogen-infected tick on the dog). The *R. parkeri*-positive tick was scored to have an engorgement score of 5, while the other ticks on this dog had engorgement scores of 0–3 (Table 3).

The majority of dogs that harbored rickettsiae-positive ticks (*n* = 6/8) had only a single positive tick. Two dogs had multiple ticks test positive for endosymbiotic rickettsiae in the subset of ticks that were tested. One dog from the neighborhood Pedro J. Mendez harbored 2 *Candidatus* R. andeanae-positive ticks among the 10 that were tested; there was a total of 88 ticks present on this dog. One 1-year-old, female, mixed-breed dog from the neighborhood 15 de Enero harbored 4 infected ticks (3 with *Candidatus* R. andeanae and 1 with *R. amblyommatis*) among the 10 that were tested (118 ticks were present on this dog).

## 4. Discussion

We documented three species of *Rickettsia* in brown dog ticks removed from owned dogs in low-income neighborhoods of Reynosa in northeastern México. In particular, we found *R. parkeri*, a pathogenic SFGR in *Rh. sanguineus*; this pathogen has not previously been detected in northern México [4,58]. This bacterium causes *Rickettsia parkeri* rickettsiosis and is most commonly transmitted by *A. maculatum* (gulf coast ticks) with similar symptomatic manifestation in humans as RMSF, but slightly less severe [59]. Human clinical diagnostic tests often cross-react between *R. rickettsii* and *R. parkeri* [59], leading to misdiagnosis.

The *R. parkeri*-infected tick was a fully engorged nymph, and direct testing of the host dog blood as well as the other 21 ticks attached to this dog yielded a negative result. Typically, rickettsiae circulate in the blood and then establish in endothelial cells of tissues such as skin and other organs [42]. The lack of *R. parkeri* found in the whole blood of the dog could represent either that this nymphal tick had acquired *R. parkeri* (i) from the previous larval blood meal; (ii) from transovarial transmission [60], (iii) that the dog had an established infection in the skin rather than circulating *R. parkeri* in the bloodstream; or (iv) the level of rickettsemia was below the limit of detection of the assay; as such, a negative blood test does not rule out canine rickettsiae infection [61]. Skin biopsy of this dog to test for *Rickettsia* spp. could further illuminate the infection status of the dog [62,63,64,65]. *Rickettsia* spp. are in the salivary glands of infected ticks and can transmit to the host as fast as 10–30 min from the onset of blood feeding [66,67].

Our survey is the first to document *Candidatus* R. andeanae from a *Rh. sanguineus* s. l. in northern México [68]. This uncultured rickettsiae is regarded as an endosymbiont [69,70,71,72]. *Candidatus* R. andeanae has been isolated from *A. maculatum* in both Perú, México, and the United States [25,26,68,69,71,73], but only documented in *R. sanguineus* sensu lato in Perú [68]. Most studies found *Candidatus* R. andeanae to be sympatric with *R. parkeri*, as we found in our samples (Table 3), or co-infecting *A. maculatum*.

We detected *R. amblyommatis*, also known as ‘*R. amblyommii*’. The pathogenicity of this species is medically undeclared, but current investigations suggest that it can be opportunistically pathogenic [74,75,76,77,78]. Studies have reported *R. amblyommatis* to cause fever in guinea pigs [77], to have been isolated from a rash of a human [79], to have been associated with some pathology in humans [75,78], and recently, to have shown load dependency to cause morbidity or mortality in mice [74]. This species is geographically widespread and usually detected in tick species that encounter humans quite frequently, with *A. americanum* serving as a vector [73,79,80].

*Rhipicephalus sanguineus* s. l. has been implicated in recent human epidemics of RMSF, in which high tick burdens on dogs were associated with human disease cases [15,27,28,29,30,31,32,36]. Our analyses did not find an interaction between Rickettsia within attached ticks and the tick burden of dogs (Table S4). In fact, the *R. parkeri*-infected tick was from a neighborhood that had one of the lowest average tick burdens on dogs of the enrolled neighborhoods (Figure 2). Furthermore, contrary to some studies, we did not find that any life history data of the dogs correlated with tick burdens [30,32,81]. Although, we did find that the mean *Rh. sanguineus* burdens did vary significantly among neighborhoods (Figure 4). Further, we found that tick burdens among dogs were aggregated, as expected based on the parasite burden literature [82]. The highest tick burden was from a neighborhood that is relatively more exposed to the forest edge (Figure 1) than some other neighborhoods. Prior studies have found that areas with high densities of free-roaming dogs and landscape factors that are often associated with poverty (e.g., presence of trash) have increased risk of RMSF [30,32]. The *R. parkeri*-positive *Rh. sanguineus* was from a dog in a neighborhood adjacent to a lagoon with the third highest average tick burden, suggesting that infection may not be able to be predicted alone by tick burdens or the exposure to the rural areas [28,29,30]. A prior study found that dogs living in homes near an agricultural canal had higher *Rh. sanguineus* tick burdens [30].

No active surveillance of rickettsiosis is currently underway across México by the Ministry of Health, although 1195 human cases of RMSF and 725 of other *Rickettsia* etiology were reported between 2016 and 2021. The signs and symptoms of these rickettsioses might be misdiagnosed with other endemic diseases in the region, such as dengue fever. Between 2016 and 2021, the six States of México that had the highest number of RMSF cases were Sonora, Chihuahua, California, Nuevo Leon, Veracruz, and Tamaulipas. Tamaulipas (the State in which our canine study occurred) reported RMSF cases in 2016–2019, with 3, 22, 47, and 11 cases per year, respectively, but no cases were reported during 2020 and 2021 [83]. We believe that the monitoring of dogs for rickettsiosis can supplement the detection of tick-borne pathogen surveillance by Mexican health authorities.

The limitations of the study include that all enrolled dogs were owned dogs and therefore may not represent the feral/stray dogs that exist in the same neighborhoods. Furthermore, not all collected ticks or dog blood was tested to conserve resources. Nonetheless, our criteria for prioritizing ticks for testing based on individual dogs’ burdens and the engorgement score for ticks may be useful for other investigations that wish to establish similar protocols for the representative testing of a subset of collected ectoparasites. Further, the sequence data in this study came from a single *Rickettsia* gene. Although we attempted up to five PCR protocols [41,47,49], the results included multiple bands of variable fragment sizes per reaction, which sequenced to dog DNA or tick DNA, suggesting that those protocols were not suited for use on engorged ticks where the host DNA is abundant.

## 5. Conclusions

In characterizing the infestation of *Rh. sanguineus* s. l. on dogs and tick infection with rickettsial species in a disadvantaged region of México, we provided evidence that the non-invasive monitoring of dogs can be utilized for the efficient detection of tick-borne pathogens. These results illustrate the value of using dogs as sentinels and highlight the potential to use dogs as key targets for vector control techniques to prevent human tick-borne disease emergence [28,29]. Recent trials suggest that warmer temperatures induce *Rh. sanguineus* s. l. to bite humans more often [84]; accordingly, canine surveillance has increasing potential to provide information critical for assessing transmission/human risk, especially in a warming climate. Long-term monitoring programs of dogs should be emphasized for the early detection of changing tick abundance and infection prevalence on dogs in northern México, which may be predictive of human disease risk.

## Figures and Tables

**Table 1:** Primers used to test for rickettsiae in this study.

Gene	Primers	Nucleotide Sequence (5′-3′)	Amplicon Size	Reference
Citrate synthase	RrCS.372 RrCS.989	TTTGTAGCTCTTCTCATCCTATGGC CCCAAGTTC CTTTAATACTTCTTTGC	617 bp	[47]
Citrate synthase	RpCs.877p RpCs.1258n	GGGGGCCTGCTCACGGCGG ATTGCAAAAAGTACAGTGAACA	381 bp	[48]
rOmpB	120-M59 120-807	CCGCAGGGTTGGTAACTGC CCTTTTAGATTACCGCCTAA	862 bp	[49,50]
OmpA	Rr190-70 Rr190-701	ATGGCGAATATTTCTCCAAAA GTTCCGTTAATGGCAGCATCT	632 bp	[51]
OmpA	Rr190-70 Rr190-701 Rr190-602	ATGGCGAATATTTCTCCAAAA GTTCCGTTAATGGCAGCATCT AGTGCAGCATTCGCTCCCCCT	550 bp	[41] (modified for touchdown PCR in this study)

**Table 2:** A collection summary of each neighborhood sampled in Reynosa, northeastern México. The table indicates all dogs enrolled in the study, their total ticks removed, the average tick burden, the dogs tick infestation prevalence, the rickettsiae prevalence, and the *Rickettsia* spp. amplified from ticks removed from each of the six neighborhoods. Overall metrics are also given.

Neighborhood	Dogs	Total Ticks	Mean Tick Burden	Dog Infestation Prevalence	Rickettsiae Prevalence of Ticks	Rickettsiae Species
15 de Enero	21	497	23.67	67% (14/21)	9.38% (6/64)	*R. amblyommii*, *R. andeanae*
Aquiles Serdán	22	84	3.82	36% (8/22)	5.88% (1/17)	*R. amblyommii*
La Cima	45	939	20.87	60% (27/45)	0	NA
Col. Margarita Maza de Juárez	9	18	2.00	67% (6/9)	16.67% (1/6)	*R. andeanae*
Pedro J. Méndez	19	347	18.26	63% (12/19)	3.45% (2/58)	*R. parkeri*, *R. andeanae*
Villa Florida	52	285	5.48	37% (19/52)	1.69% (1/59)	*R. andeanae*
Overall	168	2170	12.92	51% (86/168)	4.11 % (12/292)	

**Table 3:** Host and tick attributes for ticks infected with *Rickettsia* species from Reynosa, northeastern México.

Dog Identification	Dog Sex	Dog Age (Years)	Dog Breed	Dog Tick Burden	No. Ticks Processed	Tick Infection Prevalence	Life Stage	Sex	Engorgement	Rickettsiae
19PJMD1	F	2	Mix	22	4	25% (1/4)	N	NA	5	*R. parkeri*
19PJMD6	M	1	Mix	88	10	20% (2/10)	A	F	3	*R. andeanae*
							N	NA	4	*R. andeanae*
19MMJD01	F	2	Mix	2	1	100% (1/1)	A	F	1	*R. andeanae*
19VFD30	F	4	Chihuahua	7	1	100% (1/1)	L	NA	4	*R. andeanae*
190615DED1	M	5	Mix	75	10	10% (1/10)	A	M	NA	*R. andeanae*
1915DED10	F	1	Mix		10	40% (4/10)	A	F	3	*R. andeanae*
							A	F	2	*R. andeanae*
							N	NA	3	*R. andeanae*
							A	F	5	*R. amblyommii*
190615DED4	F	0.33	Mix	13	3	33% (1/3)	A	F	0	*R. amblyommii*
19ASD11	F	0.83	Mix	74	10	10% (1/10)	N	NA	4	*R. amblyommii*

**Table 4:** Summary of attached ticks by life stage and engorgement status. Male ticks were unfed and not scored for engorgement. Additionally, some damaged ticks were not scored for engorgement. na = not applicable.

Engorgement Score	Adult Females	Adult Males	Nymphs	Larvae	Total Ticks (%)
0	119	na	226	42	387 (18%)
1	94	na	68	11	173 (8%)
2	107	na	122	46	275 (13%)
3	64	na	180	71	315 (15%)
4	32	na	128	18	178 (8%)
5	22	na	115	20	157 (7.3%)
Engorgement not scored	13	621	32	9	675 (31%)
Total ticks	451	621	871	217	2160

## Data Availability

The data presented in this study are openly available in the Texas A&M Libraries OAKTrust Digital Repository at https://hdl.handle.net/1969.1/195898 (accessed on 2 April 2022).

## Supplementary Materials

The following supporting information can be downloaded at: https://www.mdpi.com/article/10.3390/ijerph19106249/s1.
