# Supplementary material for: Brown Dog Tick (Rhipicephalus sanguineus Sensu Lato) Infection with Endosymbiont and Human Pathogenic Rickettsia spp., in Northeastern México
Source: Int J Environ Res Public Health. 2022 May 20;19(10):6249. doi: 10.3390/ijerph19106249 (PMC9141927; doi:10.3390/ijerph19106249)
Supplement: Supplementary file 1 [file ijerph-19-06249-s001.zip › ijerph-1683630-supplementary.pdf]

Supplemental Tables

Table S1: Classification of each neighborhoods socioeconomic status with a few details describing each neighborhoods infrastructure.

Suburb	Socio-economic status	Others	Street type
15 de Enero	Low	Access to reduced resources, surrounded by undeveloped land with large patios.	Mostly unpaved streets
Aquiles Serdán	Low	One of the poorest areas of Reynosa.-Houses with little patios, most houses are close together and mostly made of wood.	Mostly unpaved streets
La Cima	Low – medium	Homes are made with non-substantial building materials to maximize budget and decrease building time, with small patios.	Paved streets
Margarita Maza de Juarez	Low – medium	Lower-middle class population; houses built with construction material, with a regular or medium-sized patio.	Paved streets
Pedro J. Mendez	Low	Access to reduced resources, surrounded by undeveloped land with large patios. This neighborhood is close to a body of water.	Mostly unpaved streets
Villa Florida	Low – medium	Homes are made with non-substantial building materials to maximize budget and decrease building time. The homes are mostly two-story and have a small patio.	Paved streets

Table S2: Predicting tick burdens on dogs. A generalized linear mixed model result of which host demographics predict host tick burden. Neighborhood as a random effect and host demographics as fixed effects. Host age is continuous, while host sex is a categorical variable, therefore, the beta coefficient for males is in comparison to females.

	Estimate	Std. Error	z value	P-value
Intercept	2.23	0.42	5.3	1.05E-07 ***
Host age (years)	-0.04	0.08	-0.47	0.637
Male hosts	0.63	0.45	1.4	0.163

Table S3: Predicting a Rickettsiae positive tick. A generalized linear mixed model* result with neighborhood as a random effect and the following: Tick life stage, tick engorgement, host tick burden, host sex, and host age, as fixed effects on the outcome of predicting a tick harboring Rickettsiae. As tick life stage is a categorical variable, larval and nymphal life stage beta coefficients are compared to the adult life stage in this regression. Similarly, as host sex is a categorical variable, the beta coefficient for males is in comparison to females. Whereas tick engorgement, host tick burden, and host age are continuous variables.

Fixed effects	Estimate	Standard Error	z value	P- value
Intercept	-1.79	0.88	-2.04	0.04 *
Larvae	-1.21	1.23	-0.99	0.32
Nymph	0.21	0.75	0.28	0.78
Tick engorgement	0.19	0.22	0.86	0.316
Tick burden of host	0.00	0.00	0.73	0.47
Male hosts	-2.06	0.87	-2.37	0.02*
Host age	-0.62	0.29	-2.1	0.03*

Table S4: Predicting if a specific neighborhood is more likely to have a Rickettsiae positive tick. A generalized linear model result with neighborhood as a fixed effect and the Rickettsiae infection status of an individual tick. Neighborhood 15 de Enero is the baseline beta coefficient or reference variable, therefore all beta coefficients below are in comparison to 15 de Enero.

Fixed effects	Estimate	Standard Error	z value	P- value
Intercept	-2.3	0.43	-5.3	1.22 e -7 ***
Aquiles Serdán	-0.6	1.1	-0.5	0.61
La Cima	-18.3	1890	-0.01	0.99
Col. Margarita Maza de Juárez	0.7	1.2	0.6	0.6
Pedro J. Méndez	-0.64	0.7	-0.9	0.38
Villa Florida	1.8	1.1	-1.6	0.10
